# Poly(I:C) Induces Distinct Liver Cell Type-Specific Responses in Hepatitis B Virus-Transgenic Mice In Vitro, but Fails to Induce These Signals In Vivo

**DOI:** 10.3390/v15051203

**Published:** 2023-05-19

**Authors:** Stefan Schefczyk, Xufeng Luo, Yaojie Liang, Martin Trippler, Mengji Lu, Heiner Wedemeyer, Hartmut H. Schmidt, Ruth Broering

**Affiliations:** 1 Department of Gastroenterology, Hepatology and Transplant Medicine, Medical Faculty, University of Duisburg-Essen, 45147 Essen, Germany; 2 Institute for Virology, University Hospital Essen, University of Duisburg-Essen, 45147 Essen, Germany; 3 Department of Gastroenterology, Hepatology and Endocrinology, Hannover Medical School, 30625 Hannover, Germany

**Keywords:** hepatitis B virus, hepatitis B surface antigen, immune evasion, pattern recognition, toll-like receptor

## Abstract

Immunopathology in hepatitis B virus (HBV) infection is driven by innate and adaptive immunity. Whether the hepatitis B surface antigen (HBsAg) affects hepatic antiviral signalling was investigated in HBV-transgenic mouse models that either accumulate (Alb/HBs, Tg[Alb1HBV]Bri44), lack (Tg1.4HBV-s-mut3) or secrete (Tg1.4HBV-s-rec (F1, Tg1.4HBV-s-mut × Alb/HBs) the HBsAg. Herein, the responsiveness of TLR3 and RIG-I in primary parenchymal and non-parenchymal liver cells was determined in vitro and in vivo. Cell type-specific and mouse strain-dependent interferon, cytokine and chemokine expression were observed by LEGENDplex™ and validated by quantitative PCR. In vitro, the hepatocytes, liver sinusoidal endothelial cells and Kupffer cells of Tg1.4HBV-s-rec mice showed poly(I:C) susceptibilities similar to the wild-type controls, while in the remaining leucocyte fraction the interferon, cytokine and chemokine induction was reduced. On the contrary, poly(I:C)-injected 1.4TgHBV-s-rec mice showed suppressed interferon, cytokine and chemokine levels in hepatocytes but increased levels in the leucocyte fraction. Thus, we concluded that liver cells of Tg1.4HBV-s-rec mice, which produce HBV particles and release the HBsAg, responded to exogenous TLR3/RIG-I stimuli in vitro but exhibited a tolerogenic environment in vivo.

## 1. Introduction

One of the most common causes of liver-related morbidity and mortality is chronic viral hepatitis caused by the hepatitis B virus (HBV) [1], with approximately 320 million people living with HBV worldwide [2]. In recent decades, HBV has been described as a stealth virus, invisible to antiviral innate immunity, due to its diverse strategies to subvert immune responses [3,4,5]. Therefore, the outcome, chronicity and pathogenesis of HBV infections are driven by complex interactions between the immune system and viral proteins.

As part of the innate immune system, toll-like receptors (TLR) and Retinoic acid-inducible gene I (RIG-I)-like receptors have the ability to recognise foreign microbial or viral patterns [6,7]. It has been demonstrated that activation of TLR pathways controls the replication of HBV [8] and activation of RIG-I leads to a suppression of HBV covalently closed circular DNA in an HBV cell culture model [9]. Although no endogenous TLR signalling is seen in HBV infection models, exogenous TLR activation is able to limit this infection. Murine models for HBV replication are sensitive to TLR-mediated interferon responses in vivo [8] and in vitro [10]. Interestingly, in HBV-transgenic mice lacking the HBV surface antigen (HBsAg), an endogenous TLR3-mediated response in a Kupffer cell (KC) has been described [11]. Furthermore, interferon-related antiviral TLR activity in human parenchymal [12] and non-parenchymal liver cells [13] is restricted to TLR3, highlighting its important role in a hepatic antiviral capacity.

Due to the limited host spectrum, animal models for HBV are not widely engaged [14]. The most commonly used animal models are mouse models, although murine hepatocytes lack the HBV entry factor (NTCP) and inefficiently form cccDNA [15]. Mouse models include HBV-transgenic strains [16,17], hydrodynamic injection models [14] and humanised liver mouse models [18]. All these models have their advantages and disadvantages. One of the HBV-transgenic models’ setbacks is the natural immune tolerance to HBV antigens, due to self-recognition [19]. However, transgenic mice exhibit competent innate immunity and are therefore an ideal model for considering intrinsic or cell-based innate immune induction and evasion.

In the present study, TLR3 and RIG-I signalling was analysed in different HBV-transgenic mouse models. The first model solely expresses the HBsAg, exhibiting ongoing HBV-related inflammation and tumour progression [20]. The second model, a 1.4-overlength model lacking the small HBsAg [17], shows endogenous, KC-mediated TLR3 activation [11]. The third model, an HBsAg-recovered 1.4-overlength model, results from the crossbreeding of models one and two [17]. This strain neither shows inflammation nor interferon responses [11], therefore representing a status of immune evasion. In the present study, hepatic TLR3 and RIG-I activation has been investigated in vitro and in vivo to describe the HBV-related immune tolerance in these models.

## 2. Materials and Methods

### 2.1. Animals

Tg(Alb1HBV)Bri44/J mice highly express large, medium and small HBsAg, designated as Alb/HBs [21]. Tg1.4HBV-S-Mut3/J mice harbour an HBV 1.4-overlength genome including a point mutation from ATG to ACC, eliminating the translational start codon of the small HBsAg gene, referred to as HBV-s-mut [17]. Crossbreeding of these two strains (HBV-s-mut × Alb/HBs) generates HBV-transgenic mice, exhibiting a recovered HBsAg expression, described as Tg1.4HBV-s-rec [11], here named HBV-s-rec. HBV-transgenic strains are on C57BL/6J background. HBV-negative littermates were used as controls, referred to as wild-type mice. Mice were bred at the University Hospital of Essen, fed *ad libitum* and received humane care according to the criteria outlined in the Guide for the Care and Use of Laboratory Animals prepared by the National Academy of Sciences and published by the National Institutes of Health. Animal breeding was approved by the local committee (*Landesamt für Natur, Umwelt und Verbraucherschutz,* LANUV Az. 81-02.04.2019.A344).

### 2.2. All-in-One Liver Cell Preparation

Primary mouse hepatocytes (PMH), liver sinusoidal endothelial cells (LSEC) and KC were prepared from 6–9-week-old wild-type and HBV-transgenic mice (Alb/HBs, HBV-s-mut and HBV-s-rec) by a two-step in situ perfusion as previously described [22]. After bead-based depletion of LSEC and KC, the final fraction was used as remaining non-parenchymal cells (rNPC). 

### 2.3. Mouse Experiments

Poly(I:C) (5 mg/kg body weight, solved in 1 mL/kg body weight PBS) or PBS (1 mL/kg body weight) was intravenously injected using 9-week-old wild-type and HBV-transgenic (Alb/HBs, HBV-s-mut and HBV-s-rec) male mice (*n* = 3). Mice were sacrificed after 24 h, serum samples were collected and PMH, LSEC, KC and rNPC were isolated. The animal experiment was approved by the local committee LANUV (Az. 84-02.04.2016.A126).

### 2.4. Cell Culture and Cell Stimulation

Cells were stimulated one day post preparation. In vitro, TLR3 stimulation was performed with 25 µg/mL poly(I:C) (high molecular weight, Invivogen, San Diego, CA, USA), whereas stimulation of RIG-I occurred through transfection of 200 ng/mL poly(I:C) using LyoVec (Invivogen) according to supplier’s protocol for 6 h and 24 h. IFNb stimulation was performed as positive control using 10 IU/mL of recombinant mouse IFNb protein (R&D Systems, Minneapolis, MN, USA). PBS was used as solvent control.

### 2.5. LEGENDplex™

Supernatants taken from PMH, LSEC, KC and rNPC 24 h after poly(I:C), IFNb (positive control) and PBS (negative control) stimulation in vitro (*n* = 3) and whole cell lysates from PMH, LSEC, KC and rNPC 24 h after poly(I:C) and PBS injection in vivo (500,000 cells lysed in 100 µL RIPA buffer) were analysed (*n* = 3). To quantify representative immune factors, LEGENDplex™ Mouse Anti-Virus Response Panel (Biolegend, San Diego, CA, USA) was performed according to manufacturer’s instructions.

### 2.6. RNA Isolation and One-Step Quantitative Reverse Transcription Polymerase Chain Reaction

According to manufacturer’s instructions, total RNA was isolated and purified using Qiazol™ solution (Qiagen, Hilden, Germany) and RNeasy Mini Kit (Qiagen). Quantitative RT-PCR was performed using QuantiFast SYBR Green RT-PCR Kit (Qiagen). Primers are given in Table 1.

### 2.7. Statistical Analysis 

Data are expressed as mean ± SD (standard deviation). Statistical significance was set at the level of *p* < 0.05. F-tests (Shapiro–Wilk normality test) and, where appropriate, unpaired *t*-tests or Welch’s *t*-tests were performed. Two-way ANOVA followed by Sidak’s multiple comparison test was performed as indicated.

## 3. Results

### 3.1. Poly(I:C) Responsiveness In Vitro Is Cell Type- and Mouse Strain-Dependent

PMH, LSEC, KC and rNPC were prepared from different HBV-transgenic mouse strains to investigate hepatic responsiveness to poly(I:C). The cells were stimulated with poly(I:C), direct treatment for TLR3 activation (25 µg/mL) or LyoVec-based transfection for RIG-I stimulation (200 ng/mL), IFNb (10 IU/mL) and PBS as the control for either 6 h or 24 h. After 6 h, the cells were lysed for quantitative RT-PCR (mean values, *n* = 5), and after 24 h, the supernatants were collected to perform a LEGENDplex™ (mean values, *n* = 3). Briefly, in both assays, the poly(I:C)-mediated interferon (IFNb and *Ifit1*), cytokine (IL10, IL1b and TNF) and chemokine (CCL2 and CCL5) responses slightly differed in all four cell fractions and in all different HBV-transgenic mouse strains.

**Figure 1:**
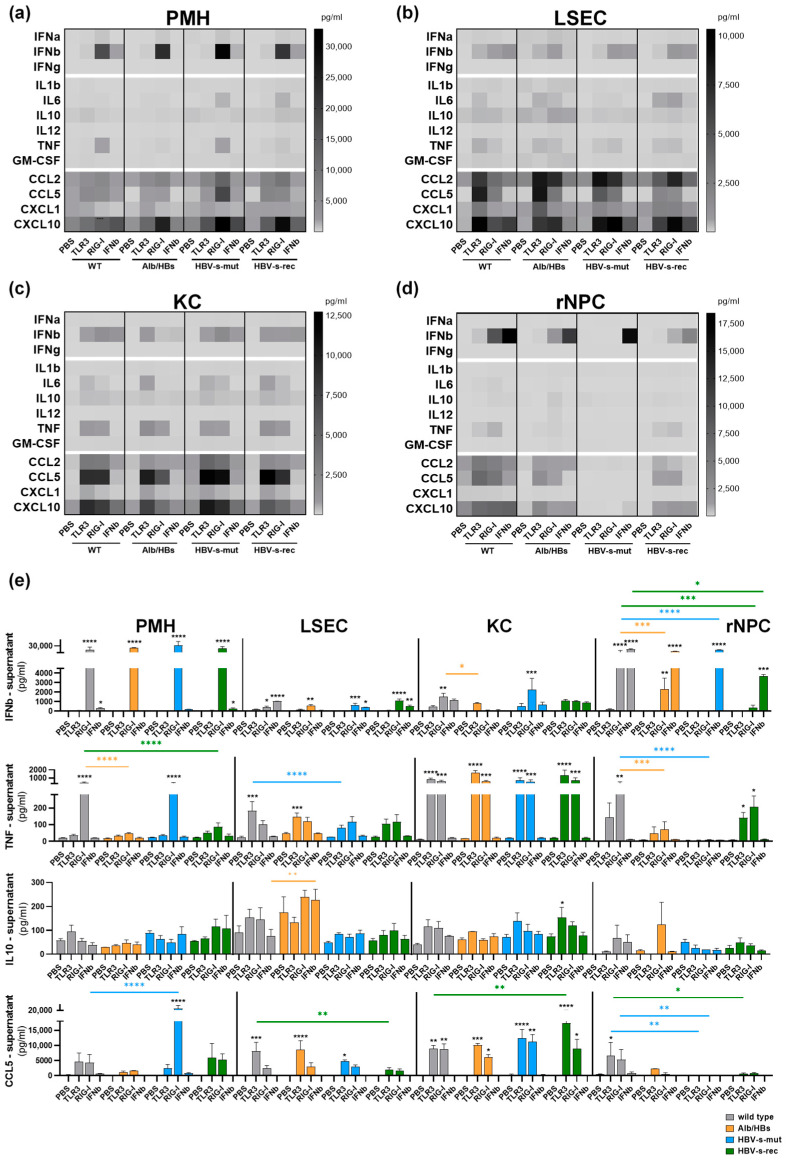
In vitro poly(I:C) responsiveness is cell type-specific in HBV-transgenic mouse strains. (**a**) PMH, (**b**) LSEC, (**c**) KC and (**d**) rNPC were isolated from 3-month-old HBV-transgenic mouse strains and stimulated with 25 µg/mL poly(I:C) for TLR3 activation, transfected using LyoVec (Invivogen) with 200 ng/mL poly(I:C) for RIG-I and treated with 10 IU/mL of IFNb or PBS as control (group sizes *n* = 3). After 24 h, supernatants were analysed using LEGENDplex™ Anti-Virus response panel. Data given as heat maps of mean values (*n* = 3) and represent protein levels (pg/mL). (**e**) Mean LEGENDplex™ signals ± SD are given for IFNb, TNF, IL10 and CCL5. Asterisks indicate significant results (* *p* < 0.05; ** *p* < 0.01; *** *p* < 0.001; **** *p* < 0.0001). PBS, phosphate-buffered saline; PMH, primary mouse hepatocytes; LSEC, liver sinusoidal endothelial cells; KC, Kupffer cells; rNPC, remaining non-parenchymal cells.

Interferons are crucial in regulating immune responses against virus infections. Downstream signals lead to the induction of IFN-stimulated genes that mediate diverse antiviral effects [24]. The LEGENDplex™ data revealed that IFNb is increased after RIG-I stimulation in the PMH and rNPC (Figure 1a,d,e), whereas in the LSEC and KC the TLR3 stimulation additionally led to IFNb secretion (Figure 1b,c,e). There was no difference in IFNb secretion in the PMH (Figure 1a,e) and LSEC (Figure 1b,e) when comparing the different mouse strains. In the KC of Alb/HBs mice, the stimulation of RIG-I was less potent in IFNb induction compared to the other mouse strains (Figure 1c,e). Interestingly, in the rNPC of HBV-s-mut mice, the stimulation of TLR3 and RIG-I did not result in IFNb secretion (Figure 1d,e). However, the rNPC of HBV-s-rec mice showed a weak secretion of IFNb in response to RIG-I activation. In conclusion, the PMH showed the strongest RIG-I-induced IFNb expression, whereas the KC and LSEC showed moderate IFNb induction after both TLR3 and RIG-I activation.

The hepatic release of pro-inflammatory cytokines leads to activation of immune cells and cytokine secretion, whereas anti-inflammatory cytokines counteract this response, thereby maintaining the immune-tolerant hepatic environment [25]. In the KC, the IL6 secretion was more potent after TLR3 stimulation (Figure 1c) than after RIG-I activation. In the LSEC, the poly(I:C)-mediated IL6 secretion was lower in the HBV-s-mut strain, compared to the other HBV-transgenic strains. In the wild-type and Alb/HBs strains, the TLR3 stimulation resulted in higher secretion of IL6 compared to the RIG-I stimulation, whereas in the HBV-s-rec strain, the RIG-I stimulation was more potent (Figure 1b). The PMH of the HBV-s-mut and HBV-s-rec mice showed an increased IL6 secretion after RIG-I stimulation. Poly(I:C)-mediated TNF secretion was reduced in the PMH of the Alb/HBs and HBV-s-rec strain (Figure 1a,e) as well as in the rNPC of the Alb/HBs and HBV-s-mut mice (Figure 1d,e). In the LSEC and KC, TNF induction was at a similar level in all HBV-transgenic strains (Figure 1b,c,e). In the rNPC, the cytokine secretion was reduced in the Alb/HBs and HBV-s-mut mice, whereas the HBV-s-rec cells secreted TNF similar to the wild-type cells (Figure 1d,e). Secretion of IL1b, IL12 and GM-CSF in response to poly(I:C) was absent in all mouse strains, except a slight measurable secretion in the LSEC of the Alb/HBs mice. Anti-inflammatory IL10 was secreted at very low levels after TLR3 stimulation in the PMH and KC, whereas IL10 secretion in the LSEC and rNPC was slightly increased after RIG-I stimulation (Figure 1a–e). Under control conditions, the IL10 secretion was increased in the PMH (Figure 1a,e) and LSEC (Figure 1b,e) of the HBV-s-mut and Alb/HBs mice, respectively. In the PMH, these levels decreased after poly(I:C) stimulation (Figure 1a,e). However, in the Alb/HBs-derived LSEC, the induction of IL10 occurred after RIG-I activation (Figure 1b,e). In the KC, IL10 secretion was at a similar level to the wild-type control in all strains (Figure 1c,e). After RIG-I stimulation, the rNPC of the Alb/HBs strain showed increased secretion of IL10, whereas the HBV-s-mut and HBV-s-rec strains were similar to the wild-type control (Figure 1d,e). To conclude, the KC were more potent than the LSEC in poly(I:C)-mediated cytokine induction. TLR3 activation was more potent in inducing cytokines than RIG-I activation. In the PMH of the Alb/HBs mice as well as the LSEC and rNPC of the HBV-s-mut mice, LEGENDplex™ only detected marginal cytokines secretion. 

Chemokines play an important role in the immune response due to the ability to recruit leucocytes to the site of infection [26]. Chemokine secretion was more strongly induced by TLR3 stimulation in the LSEC, KC and rNPC, whereas in the PMH the RIG-I stimulation tended to induce higher levels of chemokines (Figure 1a–d). The PMH from the HBV-s-mut and HBV-s-rec strains showed increased CCL2, CCL5 and CXCL10 secretion compared to the wild-type control (Figure 1a,e). TLR3-mediated CCL5 secretion was induced to a lower extent in the LSEC of the HBV-s-mut and HBV-s-rec mice. The LSEC from the HBV-transgenic strains, with the exception of the TLR3 stimulation in HBV-s-rec mice, showed increased CCL2 secretion. The CXCL10 secretion after the TLR3 stimulation was less potent in the LSEC from the Alb/HBs and HBV-s-rec mice (Figure 1b,e). In the KC, the chemokine secretion was similar between the wild-type and HBV-s-mut mice. The CCL2 secretion was also less potent in the Alb/HBs and HBV-s-re mice (Figure 1c). The poly(I:C)-mediated secretion of CCL2, CCL5 and CXCL10 in the rNPC was lower in the Alb/HBs and HBV-s-rec mice, and in the HBV-s-mut mice, the chemokine secretion was almost absent (Figure 1d,e). Generally, the LSEC and KC were strong chemokine inducers, in response to poly(I:C) (TLR3 > RIG-I). According to the cytokine profile, the rNPC of the HBV-s-mut mice only showed marginal chemokine secretion.

**Figure 2:**
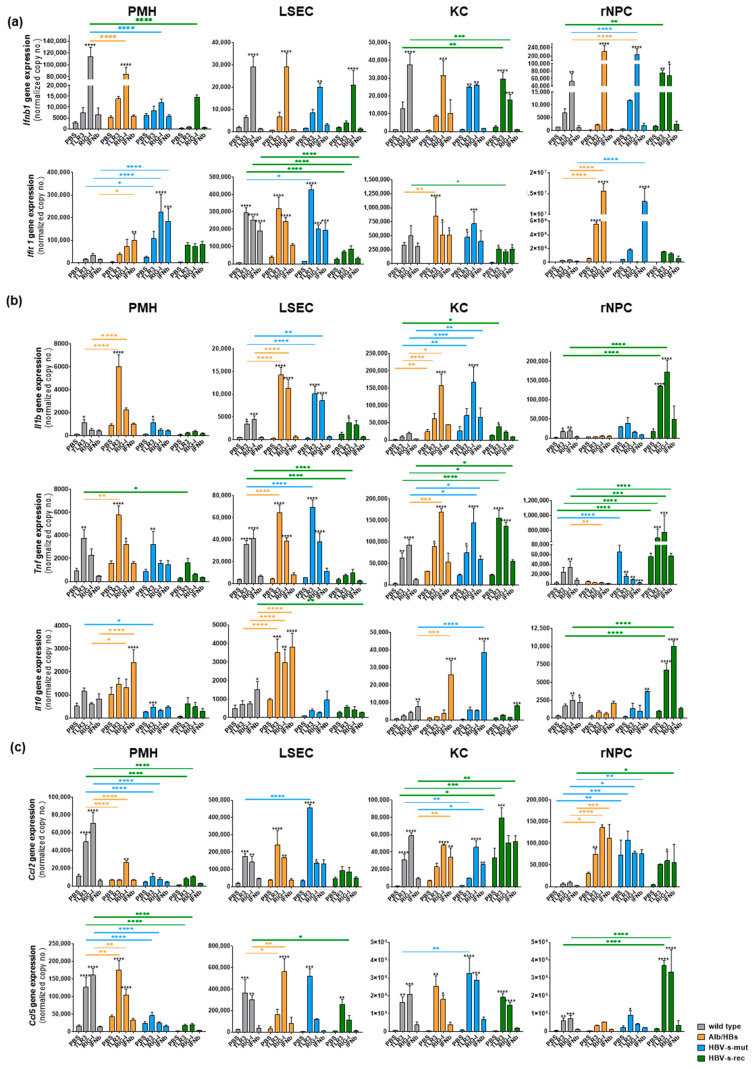

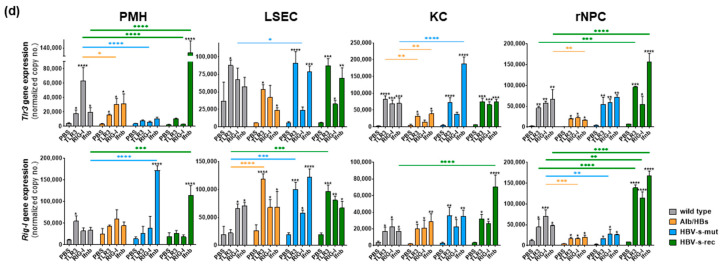
Cell type-specific responsiveness to exogenous poly(I:C) stimulation in HBV-transgenic mouse strains. After an all-in-one liver cell preparation, PMH, LSEC, KC and rNPC were isolated from wild-type, Alb/HBs, HBV-s-mut and HBV-s-rec mice and stimulated with 25 µg/mL poly(I:C) for TLR3, transfected using LyoVec (Invivogen) with 200 ng/mL poly(I:C), 10 IU/mL of IFNb or PBS as control for 6 h (*n* = 5). After 6 h, RNA was extracted afterwards and gene expression determining (**a**) interferon (*Ifnb* and *Ifit1*), (**b**) cytokine (*Il1b*, *Il10* and *Tnf*), (**c**) chemokines (*Ccl2* and *Ccl5*) responses and (**d**) *Tlr3* and *Rig-I* expression was determined by qRT-PCR. Data represent copy numbers as mean ± SD normalised to 100,000 copies of reference gene *GAPDH*. Asterisks indicate significant results (* *p* < 0.05; ** *p* < 0.01; *** *p* < 0.001; **** *p* < 0.0001). PBS, phosphate-buffered saline; PMH, primary mouse hepatocyte; LSEC, liver sinusoidal endothelial cells; KC, Kupffer cells; rNPC, remaining non-parenchymal cells.

In total, a distinct cell type- and mouse strain-dependent secretion of interferons, cytokines and chemokines was observed using LEGENDplex™. These data were validated by quantitative RT-PCR, indicating the expression of *Ifnb1*, *Ifit1* (Figure 2a), *Il1b*, *Tnf, Il10* (Figure 2b), *Ccl2* and *Ccl5* (Figure 2c). Interestingly, despite persistent inflammation in the Alb/HBs mice and a slight antiviral preactivation in the KC of the HBV-s-mut mice, the primary liver cells of the HBV-s-rec mice did not show any preactivation and responded to poly(I:C) more like the wild-type controls. With the exception of inflammatory genes (*Il1b*, *Tnf* and *Il10*), these were highly induced in poly(I:C)-treated HBV-s-rec rNPC, although LEGENDplex™ did not indicate them at the protein level. The transcriptional induction of interferon, cytokines and chemokines nicely confirmed the cell type- and mouse strain-dependent TLR3 and RIG-I responsiveness.

Of note, the magnitude of the immune induction in both assays may be influenced by the preparation procedure and experimental schedule. The basal gene expression of Tlr3 and Rig-I appeared to be the same in the different cell types and mouse strains. However, the expression in response to TLR3 and RIG-I activation as well as Ifnb stimulation (Figure 2d) appeared to be cell type- and mouse strain-dependent, suggesting a TLR3/RIG-I-dependent IRF3 and Ifnb-dependent STAT1/2 activation pattern. Poly(I:C) stimulation for 24 h, which was the subject of the present study, should only be marginally affected by the regulation in *Tlr3* and *Rig-I* gene expression. 

### 3.2. HBV-Transgenic Mouse Strains Exhibit Distinct Poly(I:C) Responsiveness In Vivo 

**Figure 3:**
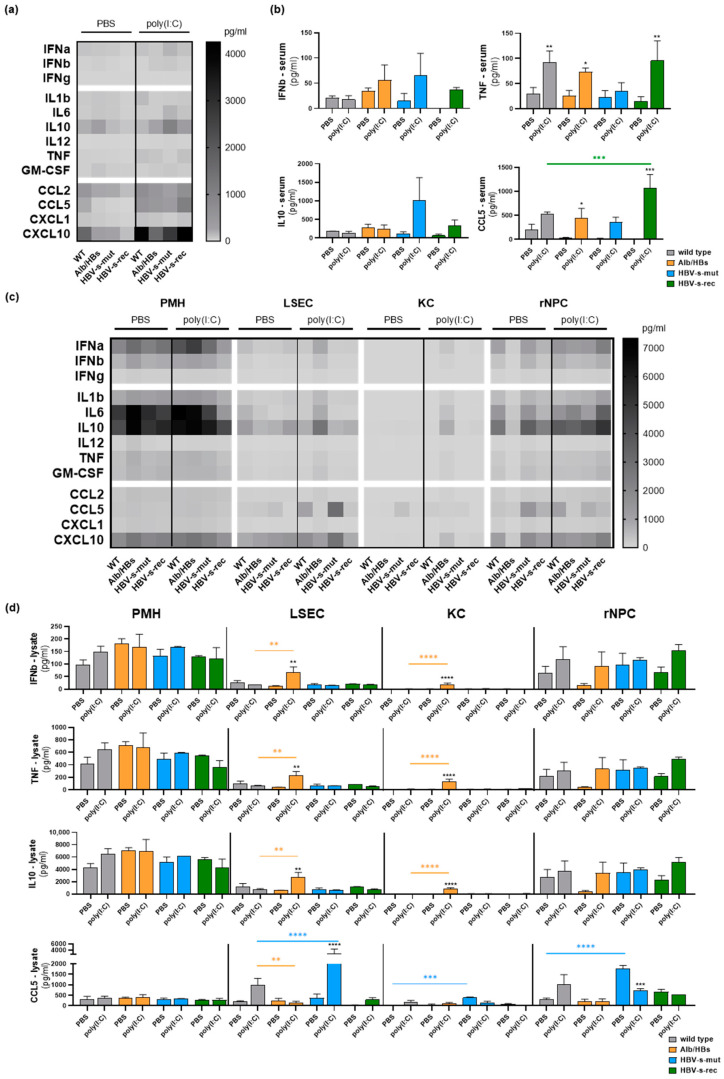
Distinct in vivo poly(I:C) responsiveness in HBV-transgenic mouse strains. The 9-week-old wild-type and HBV-transgenic mouse strains (group sizes *n* = 3) received an intravenous injection of PBS or poly(I:C) at a dosage of 5 mg/kg body weight. After 24 h, serum samples were collected and an all-in-one liver cell preparation was performed to obtain PMH, LSEC, KC and rNPC, followed by subsequent cell lysis. LEGENDplex^TM^ panel for anti-virus response was performed using (**a**) serum and (**c**) intracellular proteins. Data are given as heat maps of mean values (*n* = 3). Mean LEGENDplex™ signals ± SD are given for IFNb, TNF, IL10 and CCL5 in serum (**b**) and cell lysates (**d**). Asterisks indicate significant results (* *p* < 0.05; ** *p* < 0.01; *** *p* < 0.001; **** *p* < 0.0001). PBS, phosphate-buffered saline; PMH, primary mouse hepatocytes; LSEC, liver sinusoidal endothelial cells; KC, Kupffer cells; rNPC, remaining non-parenchymal cells.

**Figure 4:**
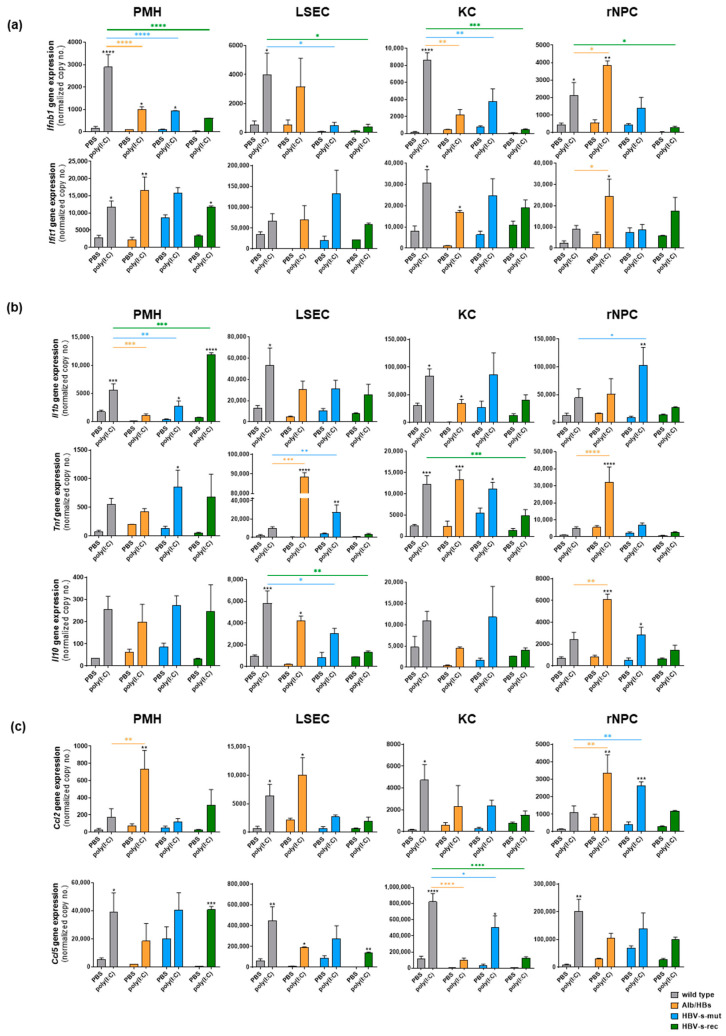
Poly(I:C) induces a cell type- and mouse strain-specific immune induction in vivo. Poly(I:C) at a dosage of 5 mg/kg body weight or PBS as control was injected via tail vein in 9-week-old wild-type, Alb/HBs, HBV-s-mut and HBV-s-rec mice, and after 24 h, an all-in-one liver cell preparation was performed to obtain PMH, LSEC, KC and rNPC (group sizes *n* = 3). Gene expression of (**a**) interferon (*Ifnb1*, *Ifit1)*, (**b**) cytokine (*IL10*, *Il1b*, *Tnf*) and (**c**) chemokine (*Ccl2*, *Ccl5*) responses was determined by qRT-PCR. Data represent copy numbers as mean ± SD normalised to 100,000 copies of reference gene *Gapdh*. PBS, phosphate-buffered saline; PMH, primary mouse hepatocyte; LSEC, liver sinusoidal endothelial cells; KC, Kupffer cells; rNPC, remaining non-parenchymal cells; WT, wild type. Asterisks indicate significant results (* *p* < 0.05; ** *p* < 0.01; *** *p* < 0.001; **** *p* < 0.0001).

The PMH, LSEC, KC and rNPC showed a specific responsiveness to TLR3 and RIG-I activation in vitro. To investigate the impact of this cell type-specific response in vivo, 9-week-old mice (group sizes *n* = 3) intravenously received 5 mg/kg body weight poly(I:C), and PBS was injected in the control group. Whether i.v. application of naked poly(I:C) only activates hepatic TLR3 or also stimulates RIG-I has not yet been described. Thus, no discrimination was made in the following in vivo setting. After 24 h, the serum samples were collected and an all-in-one liver cell preparation was performed to obtain the PMH, LSEC, KC and rNPC fractions. The cells were immediately lysed for LEGENDplex™ (Figure 3) and quantitative RT-PCR (Figure 4) analyses. A serum analysis should provide information on the basal state in HBV transgenic models as well as on the systemic effects of poly(I:C) treatment. In the PBS control groups, the LEGENDplex™ serum analysis revealed lower levels of chemokines (CCL2, CCL5 and CXCL10) in the HBV-s-rec mice and increased IL10 in the Alb/HBs mice (Figure 3a,b). However, the poly(I:C)-mediated induction of serum CCL5 and CXCL10 in the HBV-s-rec mice reached similar levels than in the wild-type controls. After poly(I:C) injection, the serum level of IL10 was slightly increased in the 1.4HBV-transgenic mice. The poly(I:C)-induced serum levels in the HBV-s-rec strain were at a similar level as in the wild-type strain, whereas the Alb/HBs and HBV-s-mut strains showed distinct serum levels of interferons, cytokines and chemokines upon poly(I:C) stimulation. The systemic effect of poly(I:C) 24 h after injection was limited to increased anti-inflammatory IL10 and the chemokines CCL5 and CXCL10.

To identify the cell type responsible for these signals, the intracellular level of proteins was determined in the PMH, LSEC, KC and rNPC of the different mouse strains using LEGENDplex™ (Figure 3c,d). An all-in-one liver cell preparation was performed 24 h after poly(I:C) injection and the cell fractions were immediately lysed (estimated time: PMH, 20 min; LSEC, 30 min; KC, 45 min; and rNPC 50–60 min postmortem). The Alb/HBs strain showed an increased intracellular level of interferons, cytokines and chemokines within the PMH under the control condition, representing the preactivation in these animals. A reduced basal level of IL6 was observed in the PMH of the PBS-treated HBV-s-rec strain, whereas the IFNa, IFNb and IL10 levels were increased. In the HBV-s-mut strain, the basal levels of the interferons, cytokines and chemokines were similar to those in the wild-type mice. The LSEC from the Alb/HBs and HBV-s-mut mice showed decreased levels of IFNa, IFNb, IL6 and IL10. On the contrary, increased basal levels of CXCL10 appeared in these mice. In the KC, no difference between the HBV-transgenic mice and wild-type control was seen, except an increased level of CCL5 in the HBV-s-mut strain. In the Alb/HBs, the injection of poly(I:C) led to increased levels of interferons and interleukins in the LSEC but a decreased level of chemokines. Except for IL6, the intracellular levels of the interferons, interleukins and chemokines were decreased in the rNPC of the Alb/HBs compared to the wild-type controls. In the HBV-s-rec mice, injection of poly(I:C) resulted in suppression of the intracellular levels of IFNa, IFNb, IL1b, IL6 and CXCL10 in the PMH and LSEC. Whereas the rNPC from this strain showed increased levels of IL1b, IL6 and IL10 in response to poly(I:C), only the CCL5 level was decreased in the rNPC. Of note, IL6 and IL10 were preactivated in the PMH and rNPC in all mice, possibly as part of the hepatic tolerogenic environment, or due to the injection and cell preparation process. The induction of interferons, cytokines and chemokines was low in the serum and liver cell lysates 24 h after TLR3/RIG-I activation in vivo, in the wild-type and HBV-transgenic mice. The liver cells of the Alb/HBs strain were most sensitive to the poly(I:C) treatment. There was no direct correlation between the serum and cellular levels of the interferons, cytokines and chemokines.

Although the poly(I:C)-induced interferon, cytokine and chemokine levels in the serum and in the different cell types were low, the gene expression analysis indicates a poly(I:C) responsiveness at least at the transcriptional level (Figure 4). In the wild-type PMH, LSEC and KC, a robust *Ifnb1* expression was induced 24 h after poly(I:C) injection. In the HBV-transgenic mice, the poly(I:C)-induced *Ifnb1* signals were significantly suppressed, except for the Alb/HBs-derived LSEC or rNPC. This indicates an in vivo responsiveness of the distinct liver cell types, on the one hand, as well as partial evasion of TLR3/RIG-I activation in the HBV-transgenic strains, on the other hand (Figure 4a). However, the response to interferon, determined by *Ifit1* expression, did not differ to that extent, neither between cell types nor mouse models. The expression of cytokines (*Il1b*, *Tnf* and *Il10*) and chemokines (*Ccl2* and *Ccl5*) after poly(I:C) injection in vivo (Figure 4b,c) was comparable to the cell type- and mouse strain-dependent responses shown in vitro (Figure 2b,c). Except for the strong cytokine induction in the PMH and the weak response in the rNPC of the HBV-s-rec mice, these appeared to be reversed. Poly(I:C) administered intravenously was able to activate TLR3/RIG-I signalling in the different liver cell populations. However, the immunosuppressive environment of the liver and post-transcriptional regulations appeared to prevent the secretion and intracellular levels of interferons, chemokines and cytokines.

**Figure 5:**
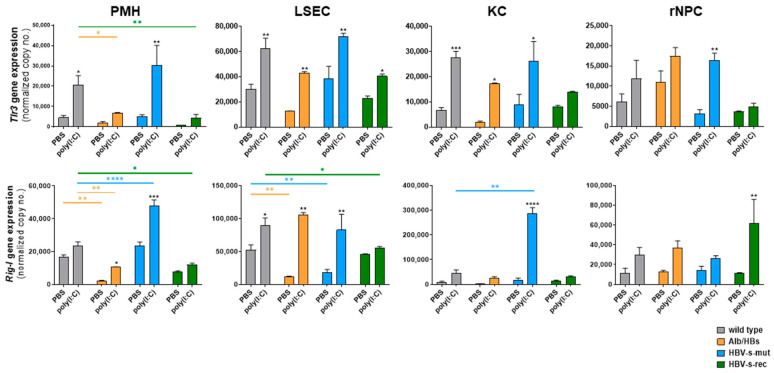
Hepatic *Tlr3* and *Rig-I* gene expression is induced by poly(I:C) injection. PBS or poly(I:C) was injected at a dosage of 5 mg/kg body weight via tail vein in 9-week-old wild-type and HBV-transgenic mice. After 24 h, an all-in-one liver cell preparation was performed to obtain PMH, LSEC, KC and rNPC (group size *n* = 3). Gene expression of *Tlr3* and *Rig-I* was determined by quantitative RT-PCR. Data represent copy numbers as mean ± SD normalised to 100,000 copies of reference gene *Gapdh*. Asterisks indicate significant results (* *p* < 0.05; ** *p* < 0.01; *** *p* < 0.001; **** *p* < 0.0001). Abbreviations: PBS, phosphate-buffered saline; PMH, primary mouse hepatocyte; LSEC, liver sinusoidal endothelial cells; KC, Kupffer cells; rNPC, remaining non-parenchymal cells; WT, wild type.

The interferon, cytokine and chemokine secretion in response to poly(I:C) in vivo was found to be less effective than in vitro and appears to be counter-regulated in the organ network. In addition to the tolerogenic environment, the distinct poly(I:C) exposure of the different cell types might also be possible. In vitro, the TLR3 and RIG-I activation induced the secretion of the selected factors in the HBV-s-rec strain, whereas the poly(I:C) treatment in vivo led to suppression of these immune factors in the PMH and LSEC, compared to the PBS control, which suggest a more tolerogenic hepatic environment. Interestingly, the rNPC fraction showed high poly(I:C) responsiveness in these mice. Therefore, the gene expression levels of *Tlr3* and *Rig-I* were determined in the PMH, LSEC, KC and rNPC 24 h after poly(I:C) and PBS injection in vivo (group sizes *n* = 3). Compared to the PBS controls, the basal expression of *Tlr3* in these cell types did not significantly differ between the wild-type and HBV-transgenic mice, except lower signals in the PMH of the HBV-s-rec mice. The poly(I:C) treatment tended to induce *Tlr3* expression, except for the rNPC of the HBV-s-rec mice (Figure 5). In contrast, the basal expression of *Rig-I* seemed to be suppressed in the Alb/HBs-derived PMH, LSEC and KC, whereas no differences occurred regarding the HBV-s-mut and HBV-s-rec strains. However, in the poly(I:C)-treated groups, the *Rig-I* expression was significantly induced in the HBV-s-mut-derived PMH and KC, while significantly lower *Rig-I* levels were seen in the PMH of the HBV-s-rec mice (Figure 5). Due to the lack of significance and low diversity, the changes seen in the gene expression of *Tlr3* and *Rig-I* are likely not involved in cell type- and mouse strain-dependent poly(I:C) responsiveness. One exception appeared for HBV-s-mut-derived KC; here, *Rig-I* expression massively increased after poly(I:C) injection (Figure 5). The preactivation of the KC in the HBV-s-mut mice appeared to sensitise them to *Rig-I* gene expression. Whether TLR3 or RIG-I activation mediated these transcriptional changes cannot be distinguished with the current data set.

## 4. Discussion

The HBV-s-rec strain was developed to gain deeper insight into a more natural type of virus–host interaction. The complementation of two HBV-transgenic strains, which display either inflammatory or interferon-based HBV-related immune activation, resulted in a mouse model with normalised, HBV-controlled hepatic immune signatures. In vitro, the PMH, LSEC and KC from this strain showed no preactivation and poly(I:C) susceptibilities similar to the wild-type mice, while in the rNPC, the interferon, cytokine and chemokine secretion was reduced. However, in vivo poly(I:C)-induced interferon, cytokine and chemokine production in HBV-s-rec mice was reduced, except for the rNPC, which strongly responded. Our study describes the HBV-s-rec strain as a valuable mouse model to study virus–host interactions that lead to evasion of endogenous HBV-related immune responses.

The innate immune system has developed defence mechanisms against pathogens, including viruses. These mechanisms are triggered by pathogen detection through different pattern recognition receptors in order to control infections. Viral infections mainly induce a type I interferon response and the release of pro-inflammatory cytokines through the activation of distinct signalling pathways [27], utilising either subfamilies of TLRs [27,28] or cytosolic RNA [29] and DNA sensors [30]. How and whether the innate immune system recognises HBV remains controversial. On the one hand, it continues to be described as a “stealth” virus [3,31], unseen by the innate immune system. On the other hand, studies show that (I) RIG-I is able to detect HBV pregenomic RNA and responds with an interferon response [32], (II) naked relaxed circular HBV DNA can be detected in a STING/cGAS-dependent manner [33], (III) TLR2 is reported to detect HBV particles [34,35] and (IV) hepatic TLR3 activation is seen in the HBV-s-mut mouse model [11]. However, HBV has evolved mechanisms to subvert these host immune responses. Events of HBV-associated immune evasion have been investigated for decades. Most of these findings have been observed in artificial cell culture models [36,37,38,39,40]. A comprehensive overview of intracellular interactions in innate immune evasion is reviewed in Ortega-Prieto et al. [41]. Finally, in patients chronically infected with HBV, hepatic impairment of innate immune responses has been described at the transcriptional level [42], suggesting a correlation to serum HBsAg levels. The current study presents a set of HBV-transgenic mice with a distinct hepatic immune pattern, allowing the analysis of HbsAg-dependent immune evasion. The Alb/HBs and HBV s-mut strains showed endogenous inflammatory and antiviral signals, respectively. In contrast, the F1 progeny showed an almost normal hepatic immune status.

Antiviral TLR signalling in murine [10] and human [13,43] liver cells is restricted to TLR3 and to some extent to TLR4 [10]. Wu et al. have visualised the anti-HBV effect of TLR3-activated PMH, LSEC, KC and murine dendritic cells [10]. In addition, the RIG-I pathway can be activated in hepatocytes, resulting in antiviral activity [44]. Sato et al. have described the anti-HBV properties of RIG-I signalling [31]. More generally, interferon-inducing PRRs have been described to suppress HBV in a murine model [8]. Here, poly(I:C) was used to activate TLR3 and RIG-I in parenchymal and non-parenchymal liver cells of HBV-transgenic mice that solely express (Alb/HBs), lack (HBV-s-mut) or secrete (HBV-s-rec) the HbsAg. Cell type-specific and mouse strain-dependent responses were observed, reflecting the inflammatory and antiviral preactivation in Alb/HBs [20,21] and HBV-s-mut mice [11], respectively. Interestingly, the PMH, LSEC and KC of the HBV-s-rec mice showed a comparable response to in vitro poly(I:C) stimulation as the wild-type animals. However, the rNPC responses in the HBV-s-rec mice markedly decreased at the protein level. Our study demonstrated that HBV-s-rec mice, which likely produced HBV particles and released an HBsAg [11,17], did not show endogenous immune activation but responded to exogenous stimuli in vitro. HBsAg and HBeAg represent the two major tolerance actors of HBV [41] that affect adaptive and innate immunity. HBeAg is an important component in viral replication and is released from infected cells. HBsAg is released either as infectious Dane particles or as sub-viral particles [45]. Intracellularly, the HBsAg and HBeAg affect the TLR signalling and suppress inflammatory signals [46,47] and anti-viral interferon responses [11,48]. The HBV-s-mut and HBV-s-rec strains, used in the present study, showed suppressed gene inductions in response to poly(I:C) in vitro and in vivo, compared to the wild-type controls, thus representing valuable models for comparison of HBeAg-dominant and HBsAg-dominant immune evasion. 

The combination of immunogenic HBV-transgenic mouse models, described in the present study, is a powerful tool to address virus–host interactions, endogenous immune responses and immune evasion in HBV replicative models. However, these models are not recommended to study antiviral treatment or adaptive immunity. For these purposes, recombinant cccDNA-transgenic or humanised models [18,49,50] and hydrodynamic injection models [14] are proposed, respectively. Although cell type-specific and mouse strain-specific responses have been described, the limitations of the present study must be noted. The extent of immune induction could be influenced by the cell preparation procedure, the cell culture conditions and the experimental schedule. Therefore, direct comparisons between cell types should be made with caution. In addition, the LEGENDplex^TM^ analysis of cell culture supernatants in vitro and serum or cell lysates in vivo are also difficult to compare. In the present study, however, the comparison was made between mouse strains under these different conditions and thus mouse strain-specific differences were described.

## 5. Conclusions

The present study demonstrated specific poly(I:C) reactivity in Alb/HBs, HBV-s-mut and HBV-s-rec mice with different types of HBV-related pathophysiology. Finally, the response to exogenous TLR3/RIG-I stimulation in HBV-s-rec-derived cells was more similar to wild-type controls in vitro, except for liver lymphocytes. Taken together, the HBV-s-rec strain has the potential to be used as a model to provide further and deeper insights into virus–host interactions and may therefore contribute to the knowledge and development of antiviral regimens in the future.

## Figures and Tables

**Table 1:** Primer sequences.

Target	Company	Order No.	Forward Primer	REVERSE PRIMER
*Tnf*	Qiagen	QT00104006		
*Il1b*	Qiagen	QT01048355		
*Il10*	Qiagen	QT00106169		
*Ccl2*	Qiagen	QT00167832		
*Ccl5*	Qiagen	QT01747165		
*Ifnb*	Qiagen	QT00249662		
*Ifit1*			5’-CTGAAATGCCAAGTAGCAAGG-3′	5′-CCAAAGGCACAGACATAAGGA-3′
*Gapdh*			5′-AAATTCAACGGCACAGTCAA-3′	5′-TCTCCATGGTGGTGAAGACA-3′
*Tlr3* *			5′-TTGTCTTCTGCACGAACCTG -3′	5′-CCCGTTCCCAACTTTGTAGA-3′
*Rig-I* *			5′-TTGCTGAGTGCAATCTCGTC-3′	5′-GTATGCGGTGAACCGTCTTT-3′
*HBx region*			5′-CCGTCTGTGCCTTCTCATCT-3′	5′-TAATCTCCTCCCCCAACTCC-3′

* Primer sequence originated from Hayashi et al. [23].

## Data Availability

Not applicable.

## Abbreviations

HBV, hepatitis B virus; HBsAg, hepatitis B surface antigen, ER, endoplasmatic reticulum; TLR, toll-like receptor; KC, Kupffer cells; PMH, primary mouse hepatocytes; LSEC, liver sinusoidal endothelial cells; rNPC, remaining non-parenchymal cells; RT-PCR, real time—polymerase chain reaction; SD, standard deviation.
